# Biodistribution of a High Dose of Diamond, Graphite, and Graphene Oxide Nanoparticles After Multiple Intraperitoneal Injections in Rats

**DOI:** 10.1186/s11671-015-1107-9

**Published:** 2015-10-12

**Authors:** Natalia Kurantowicz, Barbara Strojny, Ewa Sawosz, Sławomir Jaworski, Marta Kutwin, Marta Grodzik, Mateusz Wierzbicki, Ludwika Lipińska, Katarzyna Mitura, André Chwalibog

**Affiliations:** Department of Animal Nutrition and Biotechnology, Warsaw University of Life Sciences, Ciszewskiego 8, 02-786 Warsaw, Poland; Department of Chemical Technologies, Institute of Electronic Materials Technology, Wolczynska 133, 01-919 Warsaw, Poland; Department of Biomedical Engineering, Koszalin University of Technology, Koszalin, Poland; Department of Veterinary Clinical and Animal Sciences, University of Copenhagen, Groennegaardsvej 3, 1870 Frederiksberg, Denmark

**Keywords:** Nanoparticles, Diamond, Graphite, Graphene oxide, Rat, Biodistribution, Vascular system

## Abstract

Carbon nanoparticles have recently drawn intense attention in biomedical applications. Hence, there is a need for further in vivo investigations of their biocompatibility and biodistribution via various exposure routes. We hypothesized that intraperitoneally injected diamond, graphite, and graphene oxide nanoparticles may have different biodistribution and exert different effects on the intact organism. Forty Wistar rats were divided into four groups: the control and treated with nanoparticles by intraperitoneal injection (4 mg of nanoparticles/kg body weight) eight times during the 4-week period. Blood was collected for evaluation of blood morphology and biochemistry parameters. Photographs of the general appearance of each rat’s interior were taken immediately after sacrifice. The organs were excised and their macroscopic structure was visualized using a stereomicroscope. The nanoparticles were retained in the body, mostly as agglomerates. The largest agglomerates (up to 10 mm in diameter) were seen in the proximity of the injection place in the stomach serous membrane, between the connective tissues of the abdominal skin, muscles, and peritoneum. Numerous smaller, spherical-shaped aggregates (diameter around 2 mm) were lodged among the mesentery. Moreover, in the connective and lipid tissue in the proximity of the liver and spleen serosa, small aggregates of graphite and graphene oxide nanoparticles were observed. However, all tested nanoparticles did not affect health and growth of rats. The nanoparticles had no toxic effects on blood parameters and growth of rats, suggesting their potential applicability as remedies or in drug delivery systems.

## Background

Carbon nanoparticles (CNP) are a promising type of biomaterial for diagnostic and therapeutic applications due to their high biocompatibility and low toxicity [1, 2]. Diamond nanoparticles (DN), graphite nanoparticles (GN), and graphene oxide (GO) are receiving increasing attention in biomedical sciences for a large variety of applications, including protein immobilization, biosensors, therapeutic molecule delivery, and bioimaging.

Among the chosen nanoparticles, DN have attracted the greatest attention in in vivo experiments due to their physicochemical properties such as chemical stability, small size, large surface area, high adsorption capacity, good biocompatibility [3, 4], and easy surface functionalization for photostable fluorescent and luminescent imaging [4–7]. It has been suggested that DN may prove to be an even better drug carrier [8], imaging probe [9, 10], or implant coating [11] in biological systems compared with other carbon nanomaterials. Moreover, in vivo studies have not observed any negative effects in laboratory animals. DN administered to mice over 6 months had no harmful effects on growth, fertility, immunity, and biochemical and morphological parameters of the blood [12–14].

In vivo toxicity of GN has not been extensively investigated yet. It has been reported that graphite nanoplatelets had no genotoxic or in vivo toxic effects on *Caenorhabditis elegans* [15].

Recently, the potential toxicity of graphene in biological systems has become of great concern [16–18]. It has been demonstrated that, after intravenous injection into mice, GO accumulated in the lungs, resulting in pulmonary edema and granuloma formation [17, 18]. Intravitreal injection of GO into rabbits’ eyes affected the eyeballs’ appearance, intraocular pressure, electroretinogram, and histological parameters [19]. On the other hand, surface-functionalized graphene or GO with improved water dispersity and better stability in physiological environments appear to be much less toxic [17]. Their intrinsic physical properties such as the strong light absorption and fluorescence of functionalized GO and its nanocomposites have been utilized for photothermal therapy of cancer and as a contrast agent for in vitro and in vivo imaging [20, 21].

CNP have a high potential to be used as a drug carrier; hence, there is a need to investigate various exposure routes and confirm that a chosen platform has no toxic impact on animal models. Local administration, like the intraperitoneal route of CNP, is sparsely investigated, but seems to be safe, convenient, rapid, and causing less animal stress than intravenous administration. We hypothesized that after injection, CNP may have different biodistribution and exert different effects on the intact organism. The objective of the present study was to evaluate the biodistribution of nanoparticles in the rat model with particular emphasis on the gross pathology, vascular system, and general animal condition after multiple injections of a high dose of CNP. Presented results are the first step describing in vivo biodistribution of CNP; the next steps will include micro- and ultrastructure examination, as well as evaluations at a molecular level.

## Methods

### Nanoparticles

#### Nanoparticle Suspensions

DN and GN were obtained from Skyspring Nanomaterials (Houston, TX, USA). Both nanomaterials were produced by the explosion method and synthesized to 3–4 nm. According to the manufacturer, the purity of DN was >95 % and it had a specific surface area of ~282 m^2^/g, while the purity of GN was >93 % and it had a specific surface area of 540–650 m^2^/g. GO were prepared from graphite nanoparticles by a modified Hummers method using natural graphite flakes (purchased from Asbury Carbons, Asbury, USA) in the Institute of Electronic Materials Technology (Warsaw, Poland) [22]. In a synthesis, 5 g of graphite was added into 600 mL of H_2_SO_4_ and 67 mL of H_3_PO_4_ acid mixture. KMnO_4_ (30 g) was added in portions. The reaction was kept at 50 °C with continuous stirring for 4 h. To stop the reaction, the mixture was poured on 5 mL of deionized water, and 10 mL of H_2_O_2_ was added. Oxidized graphite was purified by sedimentation and centrifugation until concentrated suspension had pH 7. In the next step, the suspension was diluted with deionized water to 250 mL and was subjected to sonication by 500 W ultrasound processor with 75 % amplitude for 5 min. Dry powder was obtained via freeze-drying process. The diameters of the GO particles ranged from 8 to 25 nm. The physical characteristics of the nanoparticles are given in Table 1.

**Table 1:** Summary of the physical and chemical properties of diamond (DN), graphite (GN), and graphene oxide (GO) nanoparticles

	DN	GN	GO
Shape	Spherical	Spherical	Irregular
Average size (nm)	3–4	3–4	8–25
Zeta potential (mV)	−15.8 ± 0.55	12.5 ± 0.43	−8.8 ± 0.25

Shape was estimated upon analysis of transmission electron microscopy pictures. Zeta potential and average size were measured by a Zetasizer. The results are means ± standard deviation

The nanoparticle powders were suspended in sterile saline solution to a concentration of 500 mg/L and sonicated at 550 W/m^2^ for 1 h in an ultrasonic bath (Sonorex Super RK 514H, Bandelin Electronic, Germany) before each injection.

#### Visualization of Nanoparticles

**Fig. 1:**
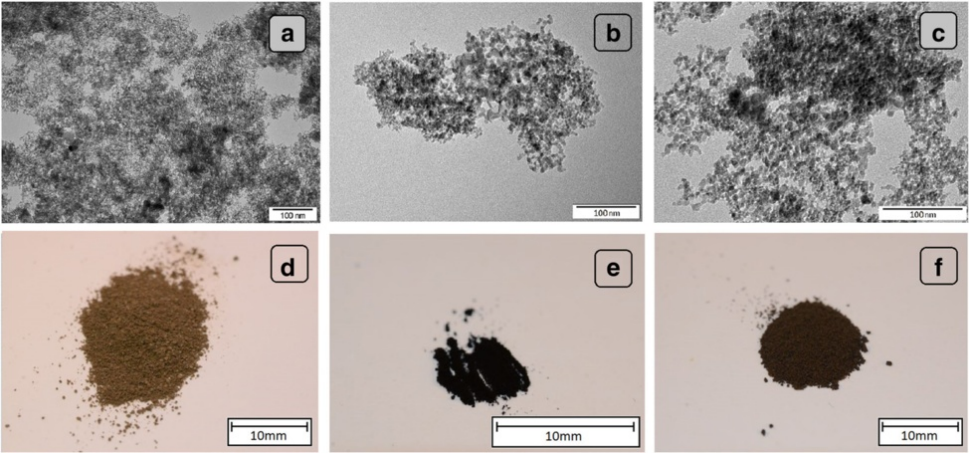
Nanoparticles visualized using transmission electron microscopy (**a**–**c**) and a digital camera (**d**–**f**). Images of diamond (**a**) and (**d**), graphite (**b**) and (**e**), and graphene oxide (**c**) and (**f**)

The size and shape of nanoparticles were inspected using transmission electron microscopy (TEM: JEM-2000EX; JEOL, Tokyo, Japan) at 80 keV. Images were captured with a Morada 11 megapixel camera (Olympus Soft Imaging Solutions GmbH, Münster, Germany). Droplets of sample solutions were placed onto Formvar-coated copper grids (Agar Scientific, Stansted, UK), and immediately after air-drying, the grids were inserted into the TEM (Fig. 1a–c). The macroscopic structure of the nanoparticle powders was visualized using a Nikon D7000 digital camera with a Nikon AF-S Micro-Nikkor 105mm f/2.8G IF-ED VR lens (Nikon, Tokyo, Japan) (Fig. 1d–f).

The DN (Fig. 1a) and GN (b) had a spherical shape. The DN powder was cinnamon brown (d), while the GN powder was the darkest and mostly fine-grained (e). The shape of GO was an irregular single layer (c), and the powder was dark brown (f).

#### Zeta Potential Measurements

The Zeta potential and size distribution of suspended nanoparticles were determined after 120 s of stabilization at 25 °C by the dynamic laser scattering-electrophoretic method with Smoluchowski approximation by Zetasizer Nano-ZS90 (Malvern, Worcestershire, UK). Each measurement was repeated three times. The mean Zeta potentials of the DN, GN, and GO solutions in 0.9 % NaCl were −15.8, 12.5, and −8.80 mV, respectively (Table 1).

### Animals

#### Animal Maintenance

The experiments were performed in accordance with Polish legal regulations concerning experiments on animals (Dz. U. 05.33.289). The experimental protocols were approved by the local ethics commission for experimentation on animals.

Forty female Wistar/cmdb outbred rats (6 weeks old, 124 ± 12 g body weight (BW)) were randomly divided into four groups (control, DN, GN, GO) and kept in polycarbonate cages with steel wire tops. They were kept under standard conditions at a room temperature of 22 ± 2 °C, 50–60 % humidity, and 12:12 light-dark cycle (lights on at 7 a.m.).

The animals had free access to water and dry pellet feed (Labofeed B standard, Wytwórnia Pasz ‘Morawski’, Poland). During the whole experiment, animal behavior and hair/skin condition were monitored.

#### Administration of Nanoparticles

Rats received multiple intraperitoneal injections of 1 mL of physiological saline (0.9 % NaCl) for the control (placebo) group and 1 mL of nanoparticle suspensions in physiological saline for the treatment groups. The injections were given for 4 weeks at 3-day intervals (eight injections in total). The nanoparticle suspensions were given at a concentration of 500 mg/L, equivalent to 4 mg of nanoparticles/kg BW.

#### Animal Euthanasia

After 4 weeks of CNP injection, the rats were fasted overnight prior to blood collection. The animals were euthanized by inhalation of isoflurane (Forane, USP, Baxter Poland). The anesthetized animals were laid in dorsal recumbency during the cardiac puncture procedure, which was used to collect blood samples from the heart. Blood was collected for evaluation of blood morphology and biochemistry parameters. Photographs of the general appearance of each rat’s interior were taken immediately after sacrifice.

#### Weight Changes and Organ Indices

Individual rats were weighed at the beginning of experiment, prior to every injection and shortly before euthanasia. The mean BW of the groups was plotted against time to reveal the course of BW gain. The rats were sacrificed and their organs were excised and weighed. The macroscopic structure of the organs was visualized using a stereomicroscope (Olympus, SZX 10, Tokyo, Japan).

#### Biochemical Serum Parameters and Hematology Measurements

Blood was collected in 5 mL tubes with a coagulation activator and 1 mL tubes with K3-EDTA. All biochemical parameters were assessed using a Miura One Clinical Chemistry Analyzer (I.S.E., Guidonia, Italy), and the reagents were purchased from Pointe Scientific Inc. (Canton, USA). Blood collected in the tubes with a coagulation activator was centrifuged at 3800 × *g* for 8 min at 4 °C for hemolysis-free serum collection. The biochemistry of the hemolysis-free serum was analyzed by standard laboratory procedures. The following parameters were examined: aspartate aminotransferase (AST), alanine aminotransferase (ALT), alkaline phosphatase (ALP), glucose, creatinine, blood urea nitrogen (BUN), total protein (TP), albumin, lactate dehydrogenase (LDH), and triglycerides (TG).

Blood collected in the tubes with K_3_-EDTA was used for hematology examination, performed with an Abacus Junior Vet Hematologic Analyzer (Diatron Group, Budapest, Hungary). The following parameters of the whole blood were examined: number of red blood cells (10^12^/L), plasma hemoglobin concentration (g/dL), hematocrit (%), number of platelets (10^9^/L), relative distribution width of the red cell population (% of covariance), red cell mean corpuscular volume (fL), mean erythrocyte hemoglobin concentration (pg), and mean cell hemoglobin concentration (g/dL). White blood cell subpopulations were quantified and expressed in absolute numbers (10^9^/L): white blood cells, lymphocyte counts, granulocyte counts, and mid cell counts. The mean platelet volume (fL) and plateletcrit/relative volume of thrombocytes (%) were also determined.

#### Data Analysis

Statistical significance was determined by one-way analysis of variance (ANOVA) and Tukey’s test using Statgraphics^®^ Plus 4.1 (StatPoint Technologies, Warrenton, VA, USA). Differences at *P* ≤ 0.05 were defined as statistically significant. All data are presented as mean ± standard deviation.

## Results

### Animals’ Body Condition/General Health Status

**Fig. 2:**
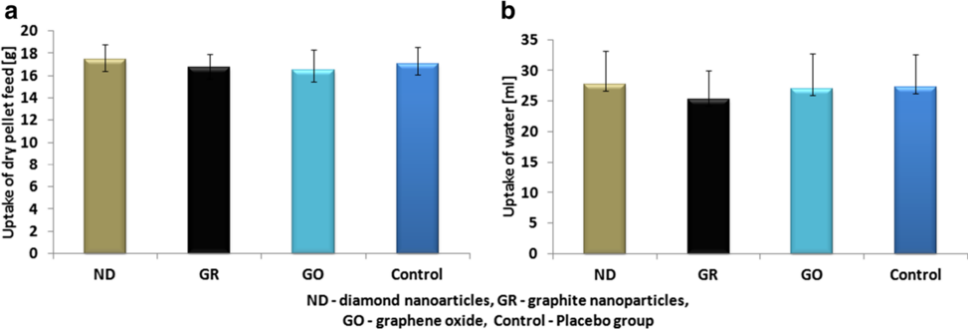
Average daily intake of dry pellet feed (**a**) and water (**b**) during 4 weeks per rat. Data presented are the average of multiple determinations (*n* = 10), with *error bars* representing the mean standard deviation

**Fig. 3:**
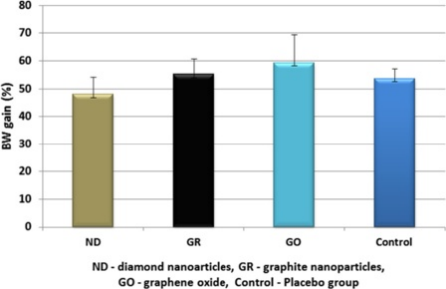
Body weight (BW) gain in rats during 4 weeks. Data presented are the average of multiple determinations (*n* = 10), with *error bars* representing the mean standard deviation

During the 4 weeks of injections, none of the animals showed physical or behavioral changes. Daily intake of feed and water and BW gain did not differ significantly between the groups (Figs. 2 and 3).

### Macroscopic Evaluation of Organs

**Fig. 4:**
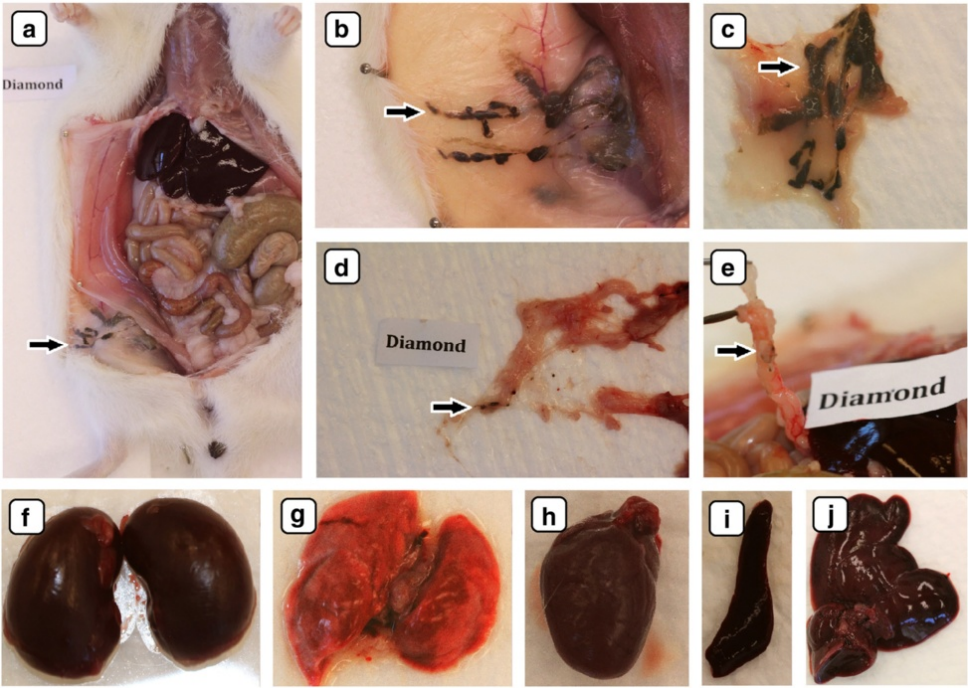
Visualization by digital camera of the biodistribution of diamond nanoparticles after multiple intraperitoneal injections into rats. Solid aggregates were lodged in injected body regions (**a**, **b**, **c**), small dots of nanoparticle aggregates were also present in abdominal lipid tissue (**e**) and mesentery (**d**). *Black arrows* indicate diamond nanoparticle aggregates. The macroscopic structure of the kidney (**f**), lungs (**g**), heart (**h**), spleen (**i**), and liver (**j**) were also examined. There were no macroscopic pathological features on the organs

**Fig. 5:**
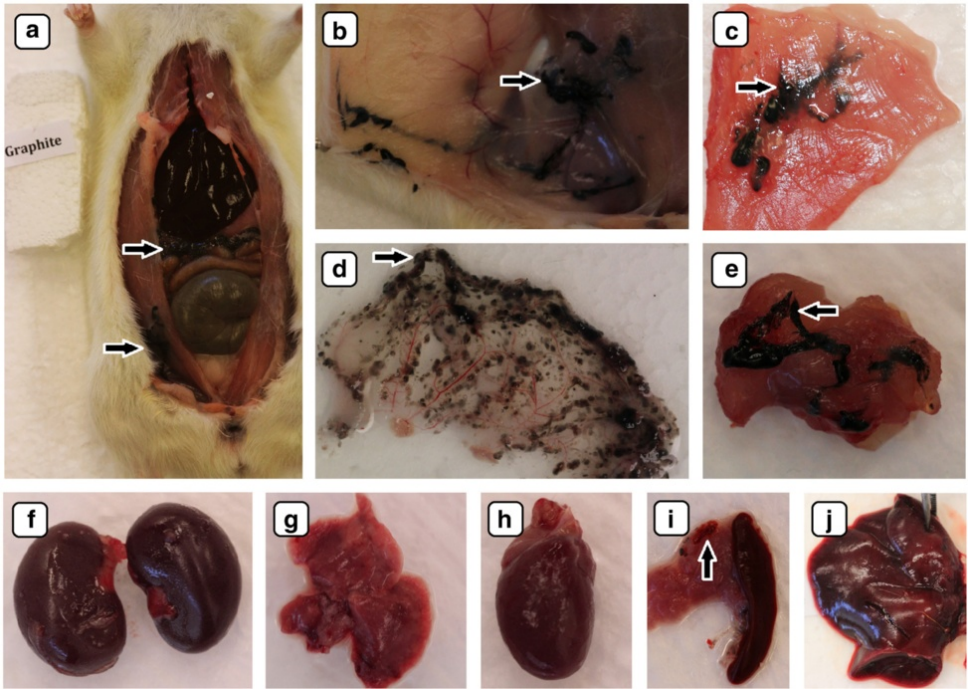
Visualization of the biodistribution of graphite nanoparticles after multiple intraperitoneal injections to rats by digital camera. Solid aggregates were placed in injection body regions (**a**, **b**, **c**, **e**) and in mesentery (**d**), small dots of nanoparticle aggregates were in lipid tissue proximity spleen serosa (**i**). *Black arrows* indicate graphite nanoparticles aggregates. The macroscopic structure of the kidney (**f**), lungs (**g**), heart (**h**), spleen (**i**), and liver (**j**) were also examined. There were no macroscopic pathological features on the organs

**Fig. 6:**
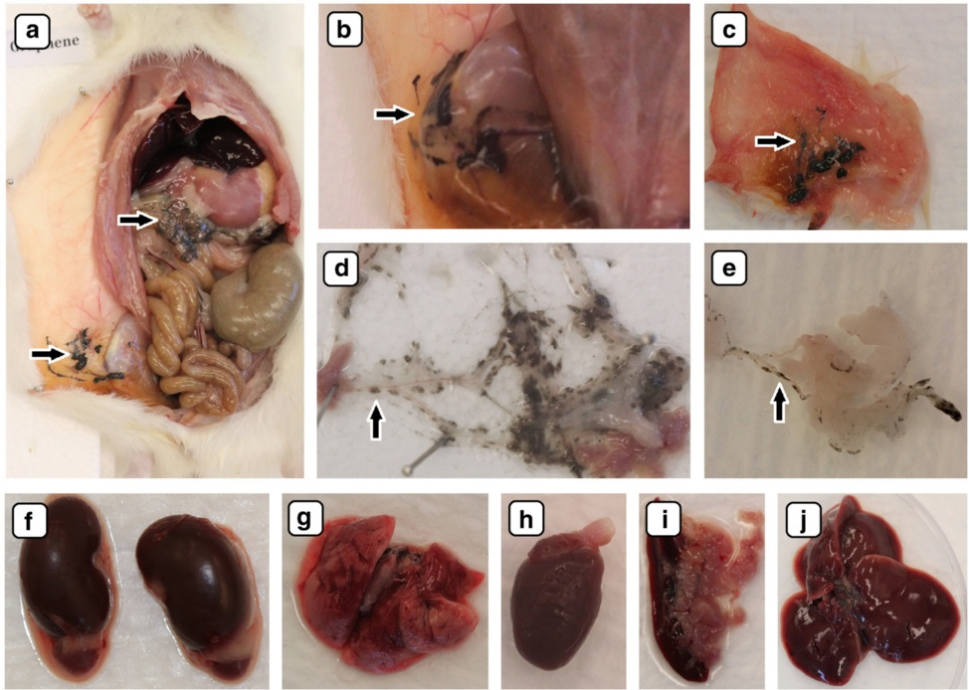
Visualization of the biodistribution of graphene oxide nanoparticles after multiple intraperitoneal injections into rats by digital camera. Solid aggregates were found in injected body regions (**a**, **b**, **c**) and mesentery (**d**). Average-sized dots of graphene oxide nanoparticle aggregates were also localized in abdominal lipid tissue (**e**). Black arrows indicate graphene oxide nanoparticle aggregates. The macroscopic structure of the kidney (**f**), lungs (**g**), heart (**h**), spleen (**i**), and liver (**j**) were also examined. There were no macroscopic pathological features on the organs

**Fig. 7:**
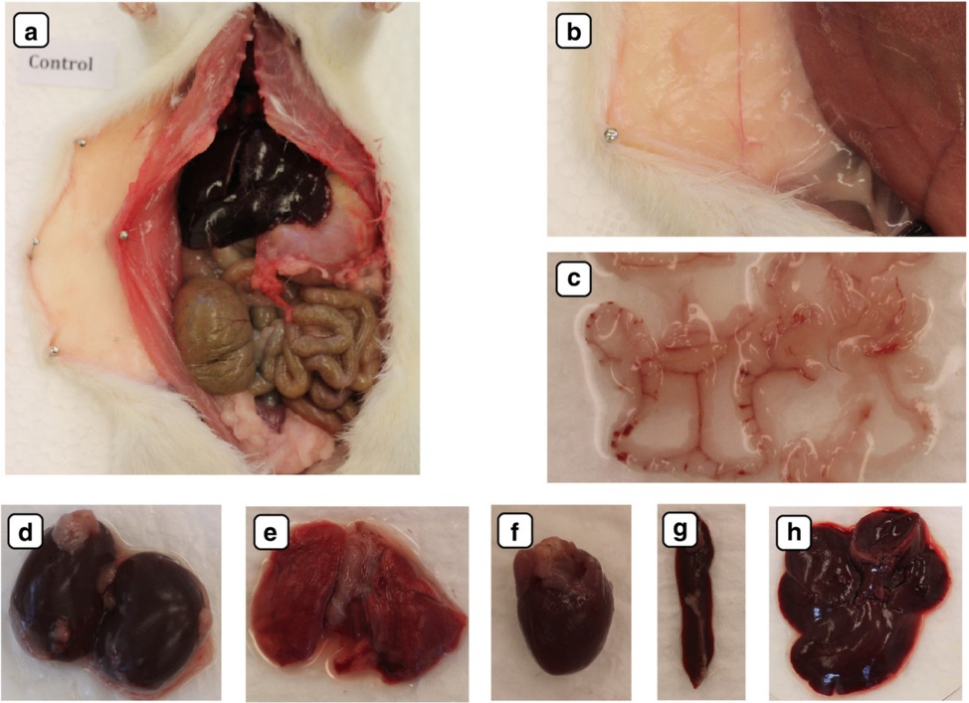
Ex vivo digital camera images of 0.9 % NaCl solution biodistribution after multiple intraperitoneal injections into rats in the placebo group. There were no pathological changes in injected body regions (**a**, **b**) or in the mesentery (**c**). The macroscopic structures of the kidney (**d**), lungs (**e**), heart (**f**), spleen (**g**), and liver (**h**) showed no toxic impact of physiological saline solution injection

During macroscopic evaluation of the organs, no pathological changes were observed (Figs. 4f–j, 5f–j, 6f–j, and 7d–h). There were no significant differences in the mean organ weights between any of the groups (Table 2).

**Table 2:** Mean organ weight (g/100 g of body weight) of the control group and groups treated with diamond (DN), graphite (GN), and graphene oxide (GO) nanoparticles

	DN	GN	GO	Control	ANOVA
					*P* value
Brain	0.83 ± 0.061	0.86 ± 0.076	0.74 ± 0.111	0.77 ± 0.117	0.494
Heart	0.34 ± 0.077	0.30 ± 0.024	0.29 ± 0.010	0.33 ± 0.020	0.405
Spleen	0.20 ± 0.028	0.25 ± 0.013	0.22 ± 0.036	0.25 ± 0.097	0.292
Kidney	0.45 ± 0.085	0.41 ± 0.043	0.41 ± 0.071	0.46 ± 0.050	0.540
Liver	4.22 ± 0.876	4.34 ± 0.427	3.95 ± 0.423	4.58 ± 0.198	0.744

The results are means of multiple determinations (*n* = 6) with ± standard deviation

**Fig. 8:**
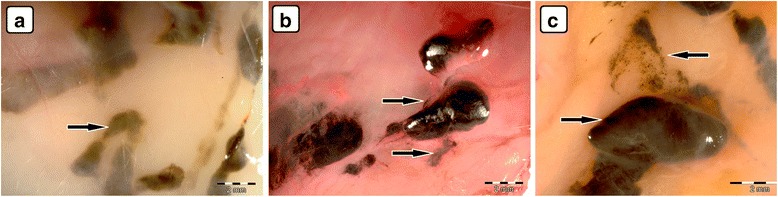
Visualization of CNP aggregates under a stereomicroscope after multiple intraperitoneal injections. Diamond (**a**), graphite (**b**), and graphene oxide (**c**) nanoparticles accumulated in a similar way; large aggregates up to 10 mm and small dots around 1 μm were formed. *Black arrows* indicate CNP aggregates

**Fig. 9:**
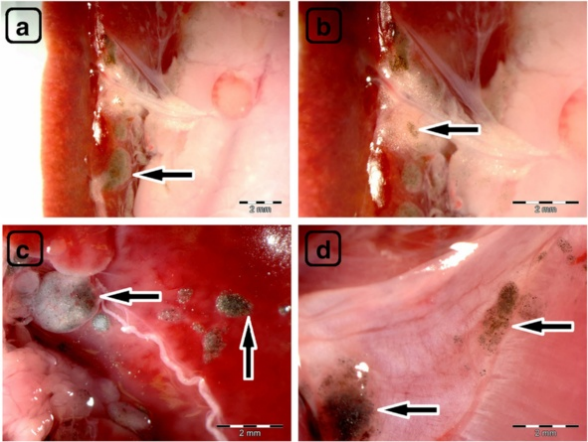
Visualization of CNP aggregates in the proximity of collected organs by stereomicroscopy. In the connective and lipid tissue of the spleen serosa, small dots (1 μm), and groups (up to 2 mm) of graphene oxide nanoparticle aggregates were formed (**a**) and (**b**). Small dots (1 μm), groups (up to 2 mm), and aggregates (up to 1 mm) of graphite nanoparticles were formed in the connective and lipid tissue of the liver serosa (**c**) and (**d**). *Black arrows* indicate CNP aggregates

Accumulation of nanoparticles was noticeable in the body tissues of all CNP-treated groups (Figs. 4a–e, 5a–e, i, 6a–e, 8, and 9). The largest solid aggregates (up to 10 mm in diameter) were in the proximity of the injection site in the stomach serous membrane, between the connective tissues of the abdominal skin, muscles, and peritoneum (Figs. 4a–c, 5a–c, 6a–c, and 8). Numerous smaller, spherical-shaped aggregates (diameters around 2 mm) were lodged among the mesentery, to a lesser extent in DN (Fig. 4d) than in the GN (Fig. 5d) and GO (Fig. 6d) groups, where the mesenteries were almost black due to their burden of nanoparticles. Smaller aggregates were observed in abdominal lipid tissue in the proximity of the injection site and mesentery (Figs. 4e, 5i, 6e, and 8). Moreover, in connective and lipid tissue in the proximity of the spleen serosa, small aggregates of GN (Fig. 5i) and GO (Fig. 9a, 9b) were observed. Most of them were small dots (<1 μm in diameter) formed in groups (around 2 mm in diameter), which could be the beginning of a process forming larger aggregates. In the proximity of connective and lipid tissue in the liver serosa, small aggregates (up to 1 mm in diameter) and dots (up to 1 μm in diameter) were observed in the GN group (Fig. 9c, d). No CNP aggregates were found in the kidneys, suggesting that CNP aggregates were unable to penetrate to retroperitoneal organs through adventitia. In the control group, there were no pathological changes surrounding the injection region (Fig. 7a, b) or among the mesentery (Fig. 7c). Moreover, the control group mesentery was much better supplied with blood and had larger blood vessels than in the CNP groups.

### Biochemical Serum Parameters and Hematology

To assess the systemic toxicity of CNP, a number of biochemical parameters in serum, such as aspartate aminotransferase (AST), alanine aminotransferase (ALT), alkaline phosphatase (ALP), blood urea nitrogen (BUN), total protein, lactate dehydrogenase, triglycerides, albumins, and creatinine were measured (Table 3). In the present study, liver function was evaluated by measuring the serum levels of AST, ALT, ALP, and creatinine. Kidney function was evaluated by analysis of the BUN and creatinine values in serum. Most of the examined biochemical parameters in blood serum did not show significant differences in comparison to the control group. However, ALP and creatinine were significantly different in the GN group compared to the control group. In all groups, the level of glucose in blood serum was significantly lower than in the control group. These results indicate that GN exposure could induce toxicological effects on the liver and kidney, but DN and GO had no effects.

**Table 3:** Mean biochemical parameters of blood serum from the control group and groups treated with diamond (DN), graphite (GN), and graphene oxide (GO) nanoparticles

	DN	GN	GO	Control	ANOVA
					*P* value
AST (IU/L)	353.7 ± 165.45	403.4 ± 169.31	363.4 ± 174.53	273.0 ± 193.34	0.861
ALT (IU/L)	89.0 ± 37.46	102.2 ± 43.10	113.5 ± 72.89	123.3 ± 70.19	0.763
ALP (IU/L)	183.1^a^ ± 42.44	285.1^b^ ± 61.57	182.8^a^ ± 25.74	175.5^a^ ± 25.08	0.000
Glucose (mg/dL)	145.7 ^b^ ± 32.89	138.1^b^ ± 19.78	144.4^b^ ± 24.59	218.8^a^ ± 56.77	0.003
Creatinine (mg/dL)	0.36^a^ ± 0.205	0.29^b^ ± 0.147	0.49^a^ ± 0.098	0.54^a^ ± 0.100	0.023
BUN (mg/dL)	49.4 ± 4.37	55.3 ± 10.16	50.9 ± 5.16	53.2 ± 11.29	0.634
TP (g/L)	57.2 ± 3.06	55.3 ± 3.33	55.3 ± 5.57	59.0 ± 5.22	0.440
Albumin (g/L)	48.5 ± 4.04	48.2 ± 3.60	49.8 ± 5.27	49.8 ± 5.19	0.882
LDH (U/L)	1496 ± 420.7	1468 ± 313.3	1516 ± 297.3	1209 ± 463.5	0.481
TG (mg/dL)	135.4 ± 34.97	99.6 ± 30.18	117.1 ± 24.63	103.4 ± 26.48	0.175

The results are means of multiple determinations (*n* = 6) with ± standard deviation

^a,b^Values with different superscripts are significantly different (*P* ≤ 0.05)

*AST* aspartate aminotransferase, *ALT* alanine aminotransferase, *ALP* alkaline phosphatase, *BUN* blood urea nitrogen, *TP* total protein, *LDH* lactate dehydrogenase, *TG* triglycerides

To assess the biocompatibility of CNP, chosen hematological parameters were measured (Table 4). The present results were compared with reference intervals for hematological parameters in diet-restricted 8 to 16-week-old Wistar rats collected under isoflurane anesthesia for female provided by Charles River Laboratories (Senneville, Canada). Most of the hematological parameters remained within the reference range, but the number of red blood cells, hemoglobin, hematocrit, and mean cell hemoglobin concentration dipped slightly when relative distribution width of the red cell population slightly exceed up.

**Table 4:** Mean hematologic parameters of the whole blood of rats

Parameters	DN	GN	GO	Placebo	ANOVA
					*P* value
White blood cells (10^9^/L)	3.01 ± 1.748	3.08 ± 1.715	2.03 ± 1.179	3.13 ± 1.496	0.586
Lymphocytes (10^9^/L)	2.65 ± 1.592	2.56 ± 1.473	1.75 ± 1.059	2.32 ± 0.665	0.622
Monocytes (10^9^/L)	0.08 ± 0.059	0.10 ± 0.049	0.06 ± 0.043	0.09 ± 0.033	0.309
Granulocytes (10^9^/L)	0.29 ± 0.207	0.43 ± 0.259	0.22 ± 0.104	0.73 ± 1.146	0.515
Red blood cells (10^12^/L)	6.67 ± 0.729	6.23 ± 0.674	5.88 ± 1.018	6.43 ± 0.813	0.440
Hemoglobin (g/dL)	11.8 ± 1.10	11.0 ± 1.29	10.7 ± 2.00	11.6 ± 1.33	0.576
Hematocrit (%)	36.30 ± 3.61	33.5 ± 3.59	32.5 ± 6.33	36.7 ± 4.06	0.392
Mean red blood cell volume (fL)	54.5 ± 2.88	54.0 ± 3.37	55.0 ± 2.10	57.2 ± 3.49	0.368
Mean cell hemoglobin (pg)	17.8 ± 0.40	17.7 ± 0.44	18.2 ± 0.40	18.1 ± 0.61	0.368
Mean cell hemoglobin concentration (g/dL)	32.7 ± 1.35	32.9 ± 1.86	33.0 ± 1.03	31.6 ± 1.18	0.336
Red blood cell distribution width (%)	15.3 ± 0.92	15.1 ± 0.82	15.0 ± 0.35	15.6 ± 1.57	0.790
Platelets (10^9^/L)	426 ± 62.7	570 ± 383.0	691 ± 127.8	513 ± 157.5	0.455
Plateletcrit (%)	0.26 ± 0.126	0.33 ± 0.181	0.63 ± 0.487	1.68 ± 2.282	0.210
Mean platelet volume (fL)	7.3 ± 0.26	6.1 ± 0.88	6.0 ± 0.98	6.4 ± 0.75	0.065

Units are in brackets. The results are means of multiple determinations (*n* = 6) with ± standard deviation

## Discussion

In the present experiment, we chose a relatively high dose (4 mg/kg BW) and multiple intraperitoneal injections (eight times) of nanoparticles to evaluate potential harmful effects on animal growth and health status. Since CNP are very small, we expected that it would be difficult to observe their distribution in animal organs by conventional microscopy, but surprisingly, DN, GN, and GO formed agglomerates that could be seen even by the naked human eye (Figs. 4a–e, 5a–e, i, 6a–e, 8, and 9). Probably because CNP are hydrophobic and aggregate readily, it is difficult to disperse them in living organisms. We did not observe any signs of inflammation, necrosis, or tissue reaction in the vicinity of injections. None of the excised organs showed any abnormalities after CNP administration (Figs. 4f–j, 5f–j, and 6f–j). It should also be noted that feed intake and body gain were not significantly different between any of the groups.

**Fig. 10:**
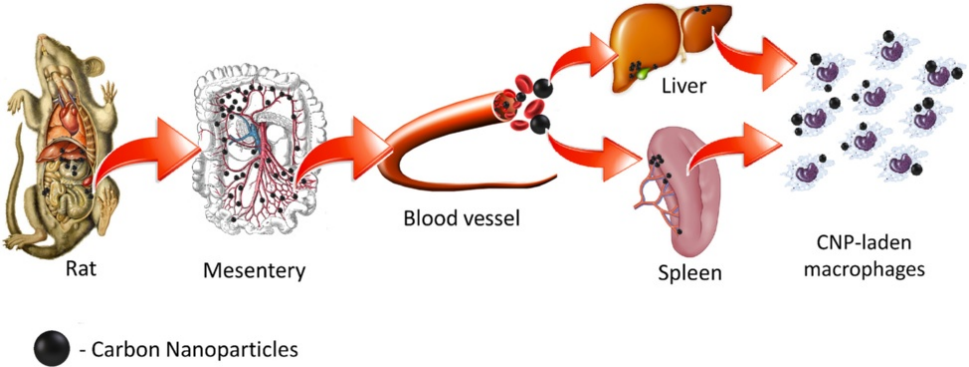
Carbon nanoparticles’ fate in vivo after intraperitoneal injection. CNP aggregates were observed in the proximity of the injection site in the stomach serous membrane and localized among the mesentery. The mesentery blood circulation system governed carbon nanoparticle trafficking and systemic translocation. The superior mesenteric vein and the splenic vein come together to form the hepatic portal vein and transport CNP to the liver and spleen, which are mononuclear phagocyte system (MPS) organs. This uptake mechanism involves macrophages in snatching up CNP from blood vessels

The majority of CNP aggregates were positioned around the injection region, while only small CNP dots were among the mesentery (DN, GO, GN) and in the proximity of the spleen (GN, GO) and liver (GN) serosa. There was no evidence of CNP being present in the kidney, lungs, heart, and their surroundings. It was previously reported that nanodiamond (ND) administered by intratracheal instillation were distributed mainly in the spleen, liver, bones, heart, and lungs [23] (Table 5). However, in mice intratracheally instilled with even a higher dose (20 mg/kg) than in our experiments (4 mg/kg), no ND were found in the liver and spleen [12]. This difference may be due to the different sources and production methods of ND. In another work by Yuan et al. [24], the distribution of ND after intravenous injection was studied in mice by using ^125^I-labeled ND. The stability of ^125^I-ND was greater than 90 % within 25 h, and the in vivo distribution showed that ND predominantly accumulated in the liver, spleen, and lung. About 60 % of the injected ND was found in the mouse liver at 0.5 h post injection, and this level stayed constant over 28 days [24]. Rojas et al. [13] labeled DN with another radionuclide, ^18^F, to study their in vivo biodistribution. The results showed that the intravenously injected DN were mainly distributed in the lung, spleen, and liver, and excreted into the urinary tract. Their research further indicated that the addition of surfactant agents did not change this distribution pattern significantly, except for a slight reduction in the urinary excretion rate of DN. It was also found that after removing DN with a larger particle size by filtration, uptake of DN was completely inhibited in the lung and spleen and significantly reduced in the liver [13]. ^60^Co-Co/graphitic-shell nanocrystals (^60^Co-Co/GC) were accumulated in mouse lung, liver, and spleen at 1, 6, 12, 18, and 24 h after intravenous injection, with the highest accumulation at 6 h but a low distribution in other tissues at all times. Moreover, ^60^Co-Co/GC began to eliminate slowly from lung and liver after 6 h, but there was a gradual increase in the spleen from 12 to 24 h [25]. However, it was reported that feeding of suspensions and microinjection of fluorescent ND into the gonads of *C. elegans* causes no harm [26]. Furthermore, there was no significant microscopic difference between the fluorescent ND-treated and control groups except for the presence of carbon-laden macrophage clusters on the peritoneal surface of the ND-treated animals [27]. Likewise, single-walled carbon nanotubes remained in the liver and spleen for over 3 months after intravenous injection [28]. Similar results and administration routes were reported for multi-walled carbon nanotubes (MWCNT). Tween 80-dispersed MWCNT accumulated in the liver and spleen with a short blood circulation time [27]. The simply dispersed MWCNT were easily recognized by macrophages and cleared from the blood very fast, and this characteristic limits their application for drug delivery [29]. In all reports of CNP biodistribution, the liver and spleen are listed as places where the nanoparticles are present but also, depending on the administration route, nanoparticles were encountered in lung, bones, and heart (Table 5). The present results also identified the liver and spleen serosa as places where nanoparticles were present, but after multiple intraperitoneal injections into rats, solid aggregates were found mainly in proximity to the injection site in the stomach serous membrane among the mesentery (Figs. 4a–c, 5a–c, 6a–c, and 8). We observed that, although CNP have good biocompatibility [30–32], they still represent a foreign non-degradable material for biological organisms. The present results and references (Table 5) indicate that the distribution of CNP in vivo involves the blood circulation for systemic translocation of CNP and for the mononuclear phagocyte system (MPS) to remove CNP from the blood. Based on the present results, we propose a scheme regarding the fate of CNP in vivo after intraperitoneal injection (Fig. 10).

**Table 5:** Summary of carbon nanoparticles in vivo accumulation with emphasis on exposure routes

Nanoparticles	Original nanoparticle name	Exposure route	Investigated animal	Nanoparticles present in organs	Reference
Graphene	PEGylated nanographene sheets wih Cy7	Intravenous	BALB/c mice	Kidney and excreted in urine	[16]
Graphene oxide	^125^I-nGO-PEG	Intraperitoneal	BALB/c mice	Liver, spleen	[17]
	^125^I-RGO-PEG				
	^125^I-nRGO-PEG				
Graphene oxide	^125^I-nGO-PEG	Intragastrically	BALB/c mice	Stomach, intestine	[17]
	^125^I-RGO-PEG				
	^125^I-nRGO-PEG				
Graphene oxide	^125^I-nGO-PEG	Intravenous	BALB/c mice	Liver, spleen	[18]
	^125^I-RGO-PEG				
	^125^I-nRGO-PEG				
Graphene oxide	Graphene oxide	Intravenous	BALB/c mice	Lungs, liver	[33]
Graphene oxide	Graphene oxide + Tween 80	Intravenous	BALB/c mice	Liver	[33]
Graphene oxide	GO	Intravitreal	Japanese white rabbit	Eyeball	[19]
Graphene oxide	^125^I-NGS-PEG	Intravenous	BALB/c mice	Liver, spleen, and excreted in urine	[39]
Graphite nanoparticles	^60^Co-Co/graphitic-shell nanocrystals	Intravenous	Kongming white mice	Liver, lungs, spleen	[25]
Graphite nanoparticles	Graphite nanoparticles	Intragastrically	Caenorhabditis elegans	Along the nematode body	[15]
Multi-walled carbon nanotubes	MWCNT	Intraperitoneal	Swiss-Webster mice	Liver	[35]
Multi-walled carbon nanotubes	^125^I-tau-MWNTs	Intravenous	Kunming mice	Liver, lungs, spleen	[27]
Multi-walled carbon nanotubes	^125^I-Tween-MWNTs	Intravenous	Kunming mice	Liver, lungs, spleen, stomach, kidney, large and small intestine	[27]
Nanodiamond	^188^Re-NDs	Intratracheal	Kun Ming mice	Bones, heart, liver, lungs, spleen	[23]
Nanodiamond	NDs-4	Intratracheal	ICR mice	Lungs	[12]
	NDs-50				
Nanodiamond	^125^I-NDs	Intravenous	ICR mice	Liver, lungs, spleen	[24]
Nanodiamond	^18^F-DNPs	Intravenous	Sprague-Dawley rats	Liver, lungs, spleen and excreted in urine	[13]
Nanodiamond	^18^F-DNPs	Intravenous	Swiss CD1 mice	Liver, lungs, spleen and excreted in urine	[13]
Nanodiamond	NDX (nanodiamond + DOX)	Intravenous	Sprague-Dawley rats BALB/c mice	Liver	[4]
Nanodiamond	DNX (nanodiamond + DOX)	Intravenous	Sprague-Dawley rats BALB/c mice	Lungs	[4]
Nanodiamond	RUDDM (Real-Dzerzhinsk ultra-disperse diamond modified)	Intravenous	Chinchilla rabbits	No aggregates but effects on biochemical blood parameters	[34]
Nanodiamond	RUDDM (Real-Dzerzhinsk ultra-disperse diamond modified)	Subcutaneous	ICR mice	Subcutaneous injection region, skin	[34]
Nanodiamond	Fluorescent nanodiamond	Gonad arms	Caenorhabditis elegans	Gonad, oocytes, early embryos	[26]
Nanodiamond	Fluorescent nanodiamond	Intragastrically	Caenorhabditis elegans	Gut (digestive track, after 20 min FND were excreted)—aggregates	[26]
Nanodiamond	Dextran-coated Fluorescent nanodiamond BSA-coated FND	Intragastrically	Caenorhabditis elegans	Intestinal cells	[26]
Single-walled carbon nanotubes	SWCNTs	Intravenous	CD-ICR mouse	Liver, lung, spleen	[28]
Single-walled carbon nanotubes	64Cu-labeled SWNT–PEG2000	Intravenous	Nude mice	Liver, kidney, spleen, intestine, lung	[36]
	SWNT–PEG5400

The blood circulation is the vehicle for CNP systemic translocation and tissue distribution. Upon administration, the blood circulation is presumably the first barrier against carbon nanoparticles, which means that blood cells have primary responsibility for governing carbon nanoparticle trafficking and systemic translocation. We investigated whether CNP could pose side effects to blood cells after intraperitoneal injection. Our data confirmed previous results by Qu et al. [33] that CNP do not exert acute toxicity on blood cells, which might be partly due to rapid clearance of nanoparticles from the circulation. However, Puzyr et al. [34] reported toxic effects on blood parameters after intravenous injection of RUDDM (Real-Dzerzhinsk ultra-disperse diamond modified) to rabbit.

The mesentery blood circulation leads to the liver, where the superior mesenteric vein and the splenic vein come together to form the hepatic portal vein. The liver is the primary organ for detoxification, defending living organisms against toxic agents; hence, it is often used in tests of the toxicity of nanomaterials [35]. Because of CNPs localizing in the liver serosa, biochemical parameters of blood serum (AST, ALT, and creatinine) reflecting hepatic injury were measured. The data obtained from the hepatotoxicity biomarker study clearly showed that GN significantly increased the activity of serum ALP and creatinine compared to the control group. However, there were no abnormalities or toxic impacts of DN and GO on rat liver. The results from the literature are conflicting. Measurements of tissue morphology and biochemical parameters in mice treated intratracheally with DN indicated dose-dependent toxicity to the lung, liver, kidney, and blood [23]. In contrast, Puzyr et al.[34] found no indications of inflammatory processes 3 months after subcutaneous exposure to RUDDM in mice, but when studied in rabbits, a number of blood biochemical parameters were affected after intravenous administration of RUDDM, suggesting that the exposure route could influence the toxic effects of RUDDM.

In the connective and lipid tissue in the proximity of the liver and spleen serosa, small aggregates of GN (Figs. 5i and 9c, d) and GO (Fig. 9a, b) were observed. As macrophages are highly concentrated in the liver and spleen, it could be possible for large numbers of GN and GO nanoparticles to accumulate in the liver and spleen serosa and in the stomach serous membrane. Such an uptake mechanism involving the mononuclear phagocyte system (MPS) is consistent with the general conception of the fate of nanoparticles in vivo [25]. The MPS recognizes nanomaterials through the binding of serum opsonin proteins to nanomaterials [33, 36]. It has been reported that carbon nanotubes were trapped by the MPS and retained mainly in the liver of mice for a long time [35, 36]. GO accumulated in the liver, with most GO aggregates localized within Kupffer cells, while no GO could be found in hepatocytes, highlighting the important role of MPS in clearing GO from the circulation [33]. CNP trapped in Kupffer cells could be excreted via bile, but the process is very slow [28].

It is thought that nanoparticles should have final hydrodynamic diameters ≤5.5 nm in order to be excreted from the rat body through the kidneys [37, 38]. In the present work, no CNP were found in the kidneys, suggesting that CNP aggregates and agglomerates were unable to penetrate the glomerular basement membrane. This might be attributed to the inability of larger particles, such as the examined nanoparticles, to cross the basement membrane. It has been demonstrated that PEGylated GO could speed up renal excretion of small marked nanoparticles [39]. Furthermore, there were no CNP aggregates in the kidneys, implying that GO could be rapidly eliminated through kidney filtration [33]. It is highly probably that single nanoparticles of DN and GN (3–4 nm) could be extracted by kidney, but larger CNP are not excreted in urine; instead, they are eliminated from the blood by the MPS and thus tend to accumulate in the spleen and liver [37, 40, 41]. Aggregation of nanoparticles could influence their ability to interact with or enter cells and thus adds complexity to the system [37]. In the present work, we did not observe the occurrence of DN, GN, or GO aggregation in the kidneys; probably, the agglomerates were not able to penetrate to retroperitoneal organs through adventitia.

## Conclusions

The tested nanoparticles had no toxic effects on general animal health status, growth, overall appearance of the animal interior, organ weight, and biochemical and hematological parameters. The CNP accumulated as small dots (<1 μm in diameter) and massive agglomerates (up to 10 mm in diameter) in proximity to the injection sites. The tendency of CNP to form agglomerates is unique and could be useful in drug delivery systems to immobilize CNP and drug complexes in the targeted body regions and then slowly release active substance.

## Abbreviations

ALP: Alkaline phosphatase
ALT: Alanine aminotransferase
AST: Aspartate aminotransferase
BUN: Blood urea nitrogen
BW: Body weight
CNP: Carbon nanoparticles
DN: Diamond nanoparticles
GN: Graphite nanoparticles
GO: Graphene oxide
LDH: Lactate dehydrogenase
MPS: Mononuclear phagocyte system
MWCNT: Multi-walled carbon nanotubes
ND: Nanodiamond particles
TEM: Transmission electron microscopy
TG: Triglycerides
TP: Total protein

## References

[1] Jastrzębska AM, Kurtycz P, Olszyna AR (2012). Recent advances in graphene family materials toxicity investigations. J Nanopart Res.

[2] Schrand AM, Hens SAC, Shenderova OA (2009). Nanodiamond particles: properties and perspectives for bioapplications. Crit Rev Solid State Mater Sci.

[3] Zhu Z, Garcia-Gancedo L, Flewitt AJ, Xie H, Moussy F, Milne WI (2012). A critical review of glucose biosensors based on carbon nanomaterials: carbon nanotubes and graphene. Sensors (Basel).

[4] Xiao J, Duan X, Yin Q, Zhang Z, Yu H, Li Y (2013). Nanodiamonds-mediated doxorubicin nuclear delivery to inhibit lung metastasis of breast cancer. Biomaterials.

[5] Krueger A, Lang D (2012). Functionality is key: recent progress in the surface modification of nanodiamond. Adv Funct Mater.

[6] Petráková V, Taylor A, Kratochvílová I, Fendrych F, Štursa J, Cígler P, Ledvina M, Fišerová A, Kneppo P (2012). Luminescence of nanodiamond driven by atomic functionalization: towards novel detection principles. Adv Funct Mater.

[7] Meinhardt T, Lang D, Dill H, Krueger A (2011). Pushing the functionality of diamond nanoparticles to new horizons: orthogonally functionalized nanodiamond using click chemistry. Adv Funct Mater.

[8] Huang H, Pierstorff E, Osawa E, Ho D (2007). Active nanodiamond hydrogels for chemotherapeutic delivery. Nano Lett.

[9] Chao J, Perevedentseva E, Chung P, Liu K, Cheng C (2007). Nanometer-sized diamond particle as a probe for biolabeling. Biophys J.

[10] Fu C, Lee H, Chen K, Lim T, Wu H, Lin P, Wei P, Tsao P, Chang H, Fann W (2007). Characterization and application of single fluorescent nanodiamonds as cellular biomarkers. PNAS.

[11] Huang H, Pierstorff E, Osawa E, Ho D (2008). Protein-mediated assembly of nanodiamond hydrogels into a biocompatible and biofunctional multilayer nanofilm. ACS Nano.

[12] Yuan Y, Wang X, Jia G, Liu J-H, Wang T, Gu Y, Yang S-T, Zhen S, Wang H, Liu Y (2010). Pulmonary toxicity and translocation of nanodiamonds in mice. Diam Relat Mater.

[13] Rojas S, Gispert JD, Martın R, Abad S, Menchon C (2011). Biodistribution of nanoparticles. In vivo studies based on 18 F radionuclide emission. ACS Nano.

[14] Liu K, Chen M, Chen P (2008). Alpha-bungarotoxin binding to target cell in a developing visual system by carboxylated nanodiamond. Nanotechnology.

[15] Zanni E, De Bellis G, Bracciale MP, Broggi A, Santarelli ML, Sarto MS, Palleschi C, Uccelletti D (2012). Graphite nanoplatelets and *Caenorhabditis elegans*: insights from an in vivo model. Nano Lett.

[16] Yang K, Zhang S, Zhang G, Sun X, Lee S, Liu Z (2010). Graphene in mice: ultrahigh in vivo tumor uptake and efficient photothermal therapy. Nano Lett.

[17] Yang K, Gong H, Shi X, Wan J, Zhang Y, Liu Z (2013). In vivo biodistribution and toxicology of functionalized nano-graphene oxide in mice after oral and intraperitoneal administration. Biomaterials.

[18] Yang K, Wan J, Zhang S, Tian B, Zhang Y, Liu Z (2012). The influence of surface chemistry and size of nanoscale graphene oxide on photothermal therapy of cancer using ultra-low laser power. Biomaterials.

[19] Yan L, Wang Y, Xu X, Zeng C, Hou J, Lin M, Xu J, Sun F, Huang X, Dai L, Lu F, Liu Y (2012). Can graphene oxide cause damage to eyesight?. Chem Res Toxicol.

[20] Yang K, Liangzhu F, Shi X, Liu Z (2013). Nano-graphene in biomedicine: theranostic applications. Chem Soc Rev.

[21] Zhang Y, Nayak TR, Hong H, Cai W (2012). Graphene: a versatile nanoplatform for biomedical applications. Nanoscale.

[22] Kurantowicz N, Sawosz E, Jaworski S, Kutwin M, Strojny B, Wierzbicki M, Szeliga J, Hotowy A, Lipińska L, Koziński R, Jagiełło J, Chwalibog A (2015). Interaction of graphene family materials with Listeria monocytogenes and Salmonella enterica. Nanoscale Res Lett.

[23] Zhang X, Yin J, Kang C, Li J, Zhu Y, Li W, Huang Q, Zhu Z (2010). Biodistribution and toxicity of nanodiamonds in mice after intratracheal instillation. Toxicol Lett.

[24] Yuan Y, Chen Y, Liu J-H, Wang H, Liu Y (2009). Biodistribution and fate of nanodiamonds in vivo. Diam Relat Mater.

[25] Zhan L, Wei Q, Yanxia G, Junzheng X, Wangsuo W (2011). Biodistribution of 60 Co–Co/graphitic-shell nanocrystals in vivo. J Nanomater.

[26] Mohan N, Chen C-S, Hsieh H-H, Wu Y-C, Chang H-C (2010). In vivo imaging and toxicity assessments of fluorescent nanodiamonds in caenorhabditis elegans. Nano Lett.

[27] Deng X, Yang S, Nie H, Wang H, Liu Y (2008). A generally adoptable radiotracing method for tracking carbon nanotubes in animals. Nanotechnology.

[28] Yang S-T, Wang X, Jia G, Gu Y, Wang T, Nie H, Ge C, Wang H, Liu Y (2008). Long-term accumulation and low toxicity of single-walled carbon nanotubes in intravenously exposed mice. Toxicol Lett.

[29] Yang S-T, Luo J, Zhou Q, Wang H (2012). Pharmacokinetics, metabolism and toxicity of carbon nanotubes for biomedical purposes. Theranostics.

[30] Zhu Y, Li J, Li W, Zhang Y, Yang X, Chen N, Sun Y, Zhao Y, Fan C, Huang Q (2012). The biocompatibility of nanodiamonds and their application in drug delivery systems. Theranostics.

[31] Fiorito S, Serafino A, Andreola F, Togna A, Togna G (2006). Toxicity and biocompatibility of carbon nanoparticles. J Nanosci Nanotechnol.

[32] Bianco A, Kostarelos K, Prato M (2011). Making carbon nanotubes biocompatible and biodegradable. Chem Commun (Camb).

[33] Qu G, Wang X, Liu Q, Liu R, Yin N, Ma J, Chen L, He J, Liu S, Jiang G (2013). The ex vivo and in vivo biological performances of graphene oxide and the impact of surfactant on graphene oxide’s biocompatibility. J Environ Sci.

[34] Puzyr AP, Baron AV, Purtov KV, Bortnikov EV, Skobelev NN (2007). Nanodiamonds with novel properties : a biological study. Diam Relat Mater.

[35] Patlolla AK, Berry A, Tchounwou PB (2011). Study of hepatotoxicity and oxidative stress in male Swiss-Webster mice exposed to functionalized multi-walled carbon nanotubes. Mol Cell Biochem.

[36] Liu Z, Cai W, He L, Nakayama N, Chen K, Sun X, Chen X, Dai H (2007). In vivo biodistribution and highly efficient tumour targeting of carbon nanotubes in mice. Nat Nanotechnol.

[37] Khan HA, Abdelhalim MAK, Al-Ayed MS, Alhomida AS (2012). Effect of gold nanoparticles on glutathione and malondialdehyde levels in liver, lung and heart of rats. Saudi J Biol Sci.

[38] Choi HS, Liu W, Misra P, Tanaka E, Zimmer JP, Itty Ipe B, Bawendi MG, Frangioni JV (2007). Renal clearance of quantum dots. Nat Biotechnol.

[39] Yang K, Wan ЌJ, Zhang ЌS, Zhang Y, Lee S, Liu Z (2011). In vivo pharmacokinetics, long-term biodistribution, and toxicology og PEGylated graphene in mice. ACS Nano.

[40] De Jong WH, Hagens WI, Krystek P, Burger MC, Sips AJ, Geertsma RE (2008). Particle size-dependent organ distribution of gold nanoparticles after intravenous administration. Biomaterials.

[41] Von Maltzahn G, Park J-H, Agrawal A, Bandaru NK, Das SK, Sailor MJ, Bhatia SN (2009). Computationally guided photothermal tumor therapy using long-circulating gold nanorod antennas. Cancer Res.

